# Predicting the risk of ibrutinib in combination with R-ICE in patients with relapsed or refractory DLBCL using explainable machine learning algorithms

**DOI:** 10.1007/s10238-025-01709-9

**Published:** 2025-05-26

**Authors:** Ni Zhu, Rong-bin Shen, Jun-fa Chen, Jian-you Gu, Si-chun Xiang, Yu Zhang, Li-li Qian, Qing Guo, Sha-na Chen, Jian-ping Shen, Jun Yan, Jing-jing Xiang

**Affiliations:** 1 Department of Hematology, The First Affiliated Hospital of Zhejiang Chinese Medical University (Zhejiang Provincial Hospital of Chinese Medicine), Hangzhou, 310006 Zhejiang China; 2 Lishui Central Hospital, The Fifth Affiliated Hospital of Wenzhou Medical University, Lishui, China; 3 Department of Hematology, Inner Mongolia International Mongolian Hospital, Hohhot, China; 4 Laboratory of Chemistry and Physics, Hangzhou Center for Disease Control and Prevention, Hangzhou (Hangzhou Health Supervision Institution), Hangzhou, 310021 Zhejiang China

**Keywords:** Diffuse large B-cell lymphoma, Machine learning, Ibrutinib, Rituximab, ICE chemotherapy

## Abstract

Relapsed or refractory diffuse large B-cell lymphoma (DLBCL) poses significant therapeutic challenges due to heterogeneous patient outcomes. This study aimed to evaluate the efficacy of the ibrutinib plus R-ICE regimen and to leverage explainable machine learning models (ML) for predicting treatment risks and outcomes. Retrospective data from 28 patients treated between March 2019 and July 2022 were analyzed. Machine learning models, including CoxBoost + StepCox, were developed and validated using bootstrap methods. Synthetic minority over-sampling combined with propensity score matching (SMOTE-PSM) addressed class imbalances. Prognostic performance was compared against the Cox proportional hazards model using decision curve and calibration analysis, as well as time-dependent ROC curves. The CoxBoost + StepCox model achieved an average C-index of 0.955 for overall survival (OS) and progression-free survival (PFS). Key prognostic indicators included elevated lactate dehydrogenase (LDH), initial treatment response, time to relapse > 12 months, and CD5 + expression. Calibration curves showed a C-index of 0.932 for OS and 0.972 for PFS in the training set. CD5 + was most predictive for OS and LDH for PFS. Machine learning models demonstrated high accuracy and clinical utility, indicating potential for data-driven treatment decisions in DLBCL.

**Supplementary Information:**

The online version contains supplementary material available at 10.1007/s10238-025-01709-9.

## Introduction

Diffuse large B-cell lymphoma (DLBCL) is a clinically heterogeneous lymphoid malignancy and the most common subtype of non-Hodgkin’s lymphoma (NHL) in adults. In the post-rituximab era, thanks to rituximab plus cyclophosphamide, doxorubicin, vincristine, and prednisone (R-CHOP), the majority of DLBCL patients can be cured. However, approximately 30–40% of patients develop relapsed or refractory (rel/ref) disease that remains the major cause of morbidity and mortality due to the limited therapeutic options, with only 10% ultimately achieving complete cure [1, 2].

Before Chimeric antigen receptor T cell (CAR-T therapy), high-dose chemotherapy followed by autologous stem cell transplantation (HDT-ASCT) was the standard treatment for chemosensitive rel/ref DLBCL [3]. CAR-T therapies, including axicabtagene ciloleucel (axi-cel) and tisagenlecleucel (tisa-cel), are innovative treatments for patients with rel/ref DLBCL [4]. A global phase 3 clinical study showed that lisocabtagene maraleucel (liso-cel) provides significant efficacy and lower toxicity as a second-line treatment for patients with primary refractory or early relapsed DLBCL, compared to standard of care SOC (3 cycles of platinum-based immunochemotherapy followed by HDT-ASCT in responders) [2]. However, not all patients are suitable for CAR-T therapy or HDT-ASCT due to individual heterogeneity, physical condition, age, economic limitations, and prior treatments received during the induction phase. Ibrutinib, a selective BTK inhibitor, hampers B-cell receptor (BCR) and nuclear factor-κB (NF-κB) pathway signaling pathways and promotes tumor cell apoptosis, and it has shown favorable tolerability and effectiveness in rel/ref DLBCL [5–7]. R-ICE, used as a salvage treatment post-R-CHOP, achieves a complete remission (CR) rate of 65% in rel/ref cases [8]. Ibrutinib in combination with R-ICE demonstrates tolerability and efficacy in rel/ref DLBCL, resulting in an overall response rate (ORR) of 90% [9].

Machine learning (ML), a subfield of artificial intelligence, has been increasingly integrated into health sciences to perform complex data analysis and predictive modeling. Common ML algorithms include decision trees, logistic regression (LR), Bayesian networks, deep learning architectures, and convolutional neural networks (CNNs) and demonstrate robust performance in clinical applications [10]. The synthetic minority over-sampling technique and propensity score matching (SMOTE-PSM) is crucial in ML and data analysis, particularly due to the limited quantity of clinical data. It effectively addresses the imbalance in clinical research datasets [11]. Time-dependent receiver operating characteristic (ROC) analysis supersedes conventional area under the curve (AUC) metrics in longitudinal studies, accommodating biomarker dynamics during follow-up [12, 13]. The calibration slope assesses the dispersion of anticipated risks and determines if they are excessively skewed, with an optimal value of 1. Calibration in the large evaluates if a model consistently overestimates or underestimates risk, with an optimal value of 0 [14]. Decision curve analysis (DCA) evaluates net benefit (NB) through threshold probability comparisons against “treat-all” and “treat-none” strategies, balancing true-positive gains against false-positive harms [15, 16].

However, to date, no research has utilized ML to predict the prognosis of ibrutinib in combination with R-ICE treatment for rel/ref DLBCL. Therefore, we employ R (version 4.2.1, https://www.r-project.org/) and ML techniques to examine the potential of this approach. The objective of this study is to investigate the efficacy of ibrutinib plus R-ICE regimen and evaluate the utility of machine learning in predicting treatment outcomes.

## Materials and methods

### Patients eligibility

A retrospective analysis was conducted on 28 patients with rel/ref DLBCL who were admitted to The First Affiliated Hospital of Zhejiang Chinese Medical University between March 1, 2019, and July 31, 2022. The primary methods employed for follow-up included telephonic interviews and in-person consultations conducted either as in-patient or out-patient reviews. The ultimate follow-up evaluation was conducted on July 31, 2022. The main biological subtypes defined in the World Health Organization 2016 classification for DLBCL are the germinal center B lymphocytes (GCB) and non-GCB subtypes which are better determined by gene expression profiling [17]. Double-expression lymphomas were defined as myelocytomatosis oncogene (MYC) expression at 40% and B-cell lymphoma 2 (BCL-2) expression at 50% of the tumor cells by immunohistochemistry [18]. Exclusion criteria included prior cancer for which the disease-free duration was less than 5 years, excluding basal cell carcinoma, cutaneous squamous cell carcinoma, carcinoma in situ of the cervix for which they received definitive treatment, major surgery within 4 weeks of study enrollment, HIV, prior serious infusion reactions, or hypersensitivity to Rituximab or etoposide or ifosfamide or nedaplatin or ibrutinib, received other clinical regiments within 30 days before the study.

### Ethical approval and informed consent

This study was approved by our institutional review board (Ethics Code: 2023-KLS-162–01). Written informed consent was obtained from all participants prior to enrollment.

### Study treatment

Three cycles of R-ICE regimen (1st day: Rituximab, 375 mg/m^2^; 2nd-4th day: etoposide, 100 mg/m^2^; 3rd day: ifosfamide, 5 g/m^2^, 24 h continuous drip; 3rd day: Nedaplatin, 1.0 g/m^2^) in combination with ibrutinib (420 mg per day) were given to the patients. Evaluation was performed after chemotherapy by positron emission tomography positron emission tomography/computed tomography (PET-CT). If CR or partial remission (PR) is achieved, followed by HDT/ASCT as patients’ intention [19].

### Follow-up and assessments

Overall survival (OS) was defined as the time interval from treatment initiation to death from any cause or last follow-up date. Patients remaining alive at database closure were censored at their last documented follow-up. Progression-free survival (PFS) was defined as the interval from the start of the study until disease progression (PD) or death for patients who achieving remission.

### Statistical analysis

Statistical analyses were performed by the R program language (version 4.2, https://www.r-project.org/), ggplot2 package (version 3.3.6) for visualizing enrichment analysis results, and SPSS statistics software (version 25, https://www.ibm.com/products/software) in this study. This study utilizes various machine learning techniques, including feature selection and model development. Key R packages such as pheatmap (version 1.0.12), gbm (version 2.1.8.1), and glmnet (version 4.1–7), among others, were employed. OS and PFS were estimated by the Kaplan–Meier method, and a comparison of survival curves was performed using the Log-Rank test. Patient characteristics and complete remission rates were compared by the X^2^ and Fisher exact tests. Two-sided *P* values and 95% confidence intervals (CI) were reported. Significance was indicated at *P* < 0.05. Toxicity was evaluated according to the common terminology criteria for adverse events version 5.0 (CTCAE v5.0).

## Results

### Data procession

**Fig. 1:**
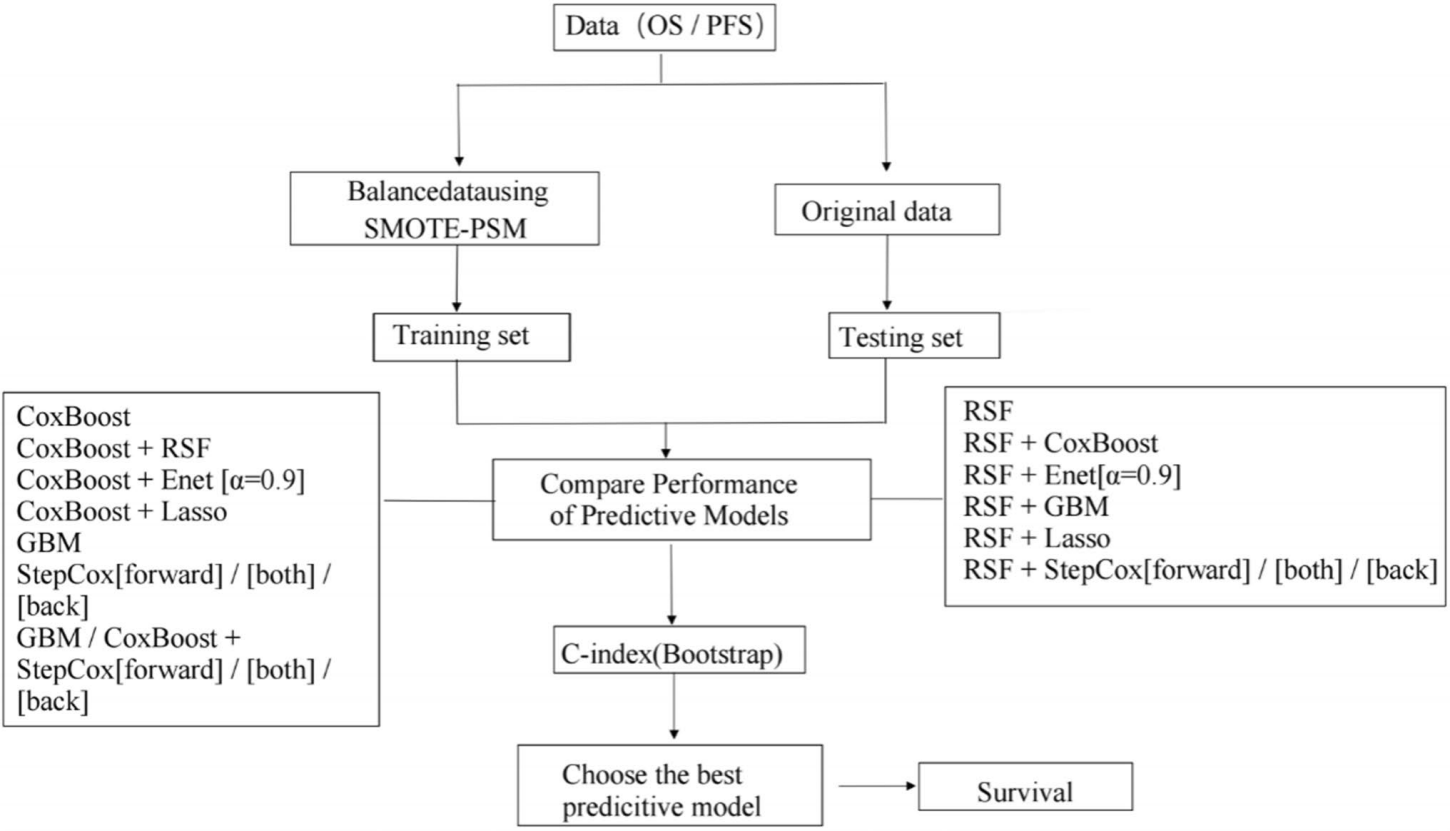
Flowchart of machine learning

The data were organized with OS and PFS designated as endpoints, and subsequently, the preliminary organized dataset (result_table) was imported and subjected to SMOTE for data augmentation. Independent variables were categorized into categorical, ordered, and numeric. The augmented dataset was then employed as a training set (result_table_PSM) for the construction of a predictive model, while the original dataset served as a testing set to evaluate the model's efficacy. The DMwR and tableone packages were utilized (sample size is small, set a smaller value) parameters: perc.ovor = bao.perc, under = 200, K = 5, Se = 0.5, and distance method as “Euclidean”. PSM was implemented using the MatchIt package with 1:1 nearest neighbor matching, a caliper width of 0.02, and survival status as the outcome variable (encoded as 0 = alive, 1 = death/PD), adjusted for gender and age. A distinctive feature of this PSM design was the 2:1 ratio of control-to-intervention group sample sizes between OS and PFS endpoints. For model validation, bootstrapping with 1,000 resamples was applied for internal validation, and the calculating Harrell’s concordance index (C-index) was calculated to quantify discriminative ability and guide feature selection (Fig. 1).

### Patient characteristics

#### Demographic characteristics, clinical features, and laboratory parameters of the training and testing sets of OS

In the testing cohort of 28 rel/ref DLBCL patients treated with ibrutinib plus R-ICE, the majority were female (57.14%) with a median age of 66.5 years (range 35–81). The median follow-up period was 30 months (range 8–40). With a median follow-up of 30 months (range 8–40), 16 patients (57.1%) achieved CR and 3 (10.7%) PR, yielding an ORR of 67.86%. Three CR patients underwent subsequent HDT/ASCT. Within this cohort, elevated LDH levels (*P* = 0.015), anemia (*P* = 0.046), CD5 + (*P* < 0.001), initial treatment response (*P* = 0.006), and time to relapse > 12 months from the initiation of R-CHOP (*P* = 0.001) were identified as adverse prognostic factors affecting OS. In the training cohort, performance status as assessed by the Eastern Cooperative Oncology Group (ECOG) (*P* = 0.024), elevated LDH (*P* < 0.001), albumin (ALB) (*P* = 0.002), β2-microglobulin (β2-MG) (*P* < 0.001), GCB subtype (*P* = 0.042), > 2 extranodal involvement sites (*P* = 0.045), the National Comprehensive Cancer Network International Prognostic Index (NCCN-IPI) (*P* = 0.001) is easy to apply and more powerful than the IPI for predicting survival in the rituximab era [20], double-expression (*P* = 0.004), CD5 + (*P* < 0.001), initial treatment response (*P* < 0.001), and time to relapse > 12 months (*P* < 0.001) were identified as adverse factors affecting OS. The baseline characteristics are detailed in Table 1.

**Table 1:** Demographic characteristics, clinical features, and laboratory parameters of the training and testing sets of OS

Variables	Training set (n)		*P*	Testing set (n)		*P*
	PFS (33)	PD (19)		PFS (19)	PD (9)	
Male, n (%)	9 (27.3)	9 (47.4)	0.244			0.350
Female, n (%)	24 (72.7)	10 (52.6)		12 (63.2)	4 (44.4)	
Mean Age years, (SD)	67.45 (5.96)	65.21 (5.50)	0.185	62.68 (12.59)	68.00 (8.67)	0.265
PS, ECOG, (SD)	1.55 (1.03)	1.84 (0.50)	0.247	1.58 (1.07)	1.78 (0.83)	0.629
Ann Arbor stage, n (%)			0.381			0.845
I				1 ( 5.3)	0 ( 0.0)	
II	0 ( 0.0)	1 ( 5.3)		3 (15.8)	1 (11.1)	
III	5 (15.2)	2 (10.5)		5 (26.3)	2 (22.2)	
IV	28 (84.8)	16 (84.2)		10 (52.6)	6 (66.7)	
B symptoms, n (%)	6 (18.2)	10 (52.6)	0.023*	7 (36.8)	5 (55.6)	0.350
Mean LDH, (SD) (U/L)	263.18 (162.56)	647.42 (267.38)	< 0.001*	310.95 (182.73)	653.67 (431.93)	0.006*
ALB, (SD) (g/L)	38.46 (1.99)	37.02 (2.63)	0.030*	37.65 (3.32)	36.48 (3.75)	0.409
β2-MG > 3 mg/L, n (%)	12 (36.4)	8 (42.1)	0.909	6 (31.6)	5 (55.6)	0.225
Anemia, n (%)	15 (45.5)	8 (42.1)	1.000	5 (26.3)	5 (55.6)	0.132
GCB, n (%)	5 (15.2)	4 (21.1)	0.872	6 (31.6)	2 (22.2)	0.609
Extranodal site (> 2), n (%)	11 (33.3)	14 (73.7)	0.012*	8 (42.1)	6 (66.7)	0.225
NCCN-IPI, n (%)			0.017*			0.141
1				1 (5.3)	0 (0.0)	
2				2 (10.5)	0 (0.0)	
3	12 (36.4)	0 (0.0)		5 (26.3)	0 (0.0)	
4	10 (30.3)	6 (31.6)		5 (26.3)	3 (33.3)	
5	7 (21.2)	9 (47.4)		2 (10.5)	4 (44.4)	
6	4 (12.1)	4 (21.1)		4 (21.1)	1 (11.1)	
7				0 (0.0)	1 (11.1)	
Ki-67 ≥ 70%, n (%)	31 (93.9)	16 (84.2)	0.511	17 (89.5)	8 (88.9)	0.963
Double-Expression, n (%)	21 (63.6)	14 (73.7)	0.662	13 (68.4)	7 (77.8)	0.609
CD5 + , n (%)	5 (15.2)	12 (63.2)	0.001*	4 (21.1)	6 (66.7)	0.019*
Initial response CR/CRU + PR, n (%)	32 (97)	11 (42.1)	< 0.001*	18 (94.7)	4 (44.4)	0.002*
Time to relapse > 12mo, n (%)	30 (90.9)	1 (5.3)	< 0.001*	17 (89.5)	2 (22.2)	< 0.001*

LDH: 109-245U/L, hemoglobin: female: 115–150 g/L, male:130-175 g/L, anemia: below reference range

^*^Significance was indicated in two-sided *p* values < 0.05

#### Demographic characteristics, clinical features, and laboratory parameters of the training and testing sets of PFS

In the training set, adverse factors impacting PFS include the presence of B symptoms (*P* = 0.023), elevated LDH (*P* < 0.001), ALB (*P* = 0.030), extranodal site > 2(*P* = 0.012), High-risk NCCN-IPI (*P* = 0.017), CD5 + (*P* = 0.001), initial response (*P* < 0.001), and time to relapse > 12 months (*P* < 0.001). In the testing set, elevated elevated LDH (*P* = 0.006), CD5 + (*P* = 0.019), initial response (*P* = 0.002), and a time to relapse > 12 months (*P* < 0.001) are identified as adverse factors affecting PFS, as indicated in Table 2. In conclusion, elevated LDH, CD5 + , initial treatment response, and time to relapse > 12 months are adverse factors affecting both OS and PFS.

**Table 2:** Demographic characteristics, clinical features, and laboratory parameters of the training and testing sets of PFS

Variables	Training set (n)		*P*	Testing set (n)		*P*
	0 (33)	1 (19)		0 (19)	1 (9)	
Male, n (%)	11 (27.3)	9 (47.4)	0.244			0.350
Female, n (%)	24 (72.7)	10 (52.6)		12 (63.2)	4 (44.4)	
Mean Age years, (SD)	67.45 (5.96)	65.21 (5.50)	0.185	62.68 (12.59)	68.00 (8.67)	0.265
PS, ECOG, (SD)	1.55 (1.03)	1.84 (0.50)	0.247	1.58 (1.07)	1.78 (0.83)	0.629
Ann Arbor stage, n (%)			0.381			0.845
I				1 ( 5.3)	0 ( 0.0)	
II	0 ( 0.0)	1 ( 5.3)		3 (15.8)	1 (11.1)	
III	5 (15.2)	2 (10.5)		5 (26.3)	2 (22.2)	
IV	28 (84.8)	16 (84.2)		10 (52.6)	6 (66.7)	
B symptoms, n (%)	6 (18.2)	10 (52.6)	0.023*	7 (36.8)	5 (55.6)	0.350
Mean LDH, (SD) (U/L)	263.18 (162.56)	647.42 (267.38)	< 0.001*	310.95 (182.73)	653.67 (431.93)	0.006*
ALB, (SD) (g/L)	38.46 (1.99)	37.02 (2.63)	0.030*	37.65 (3.32)	36.48 (3.75)	0.409
β2-MG > 3 mg/L, n (%)	12 (36.4)	8 (42.1)	0.909	6 (31.6)	5 (55.6)	0.225
Anemia, n (%)	15 (45.5)	8 (42.1)	1.000	5 (26.3)	5 (55.6)	0.132
GCB, n (%)	5 (15.2)	4 (21.1)	0.872	6 (31.6)	2 (22.2)	0.609
Extranodal site (> 2), n (%)	11 (33.3)	14 (73.7)	0.012*	8 (42.1)	6 (66.7)	0.225
NCCN-IPI, n (%)			0.017*			0.141
1				1 (5.3)	0 (0.0)	
2				2 (10.5)	0 (0.0)	
3	12 (36.4)	0 (0.0)		5 (26.3)	0 (0.0)	
4	10 (30.3)	6 (31.6)		5 (26.3)	3 (33.3)	
5	7 (21.2)	9 (47.4)		2 (10.5)	4 (44.4)	
6	4 (12.1)	4 (21.1)		4 (21.1)	1 (11.1)	
7				0 (0.0)	1 (11.1)	
Ki-67 ≥ 70%, n (%)	31 (93.9)	16 (84.2)	0.511	17 (89.5)	8 (88.9)	0.963
Double-Expression, n (%)	21 (63.6)	14 (73.7)	0.662	13 (68.4)	7 (77.8)	0.609
CD5 + , n (%)	5 (15.2)	12 (63.2)	0.001*	4 (21.1)	6 (66.7)	0.019*
Initial response CR/CRU + PR, n (%)	32 (97)	11 (42.1)	< 0.001*	18 (94.7)	4 (44.4)	0.002*
Time to relapse > 12mo, n (%)	30 (90.9)	1 (5.3)	< 0.001*	17 (89.5)	2 (22.2)	< 0.001*

LDH: 109-245U/L, hemoglobin: female: 115–150 g/L, male: 130-175 g/L, anemia: below reference range, the value of 0 indicates PFS, while the value of 1 represents PD

^*^Significance was indicated in two-sided *p* values < 0.05

### Evaluation of the model

#### Machine learning-based integrative procedure and compare performance of predictive models

**Fig. 2:**
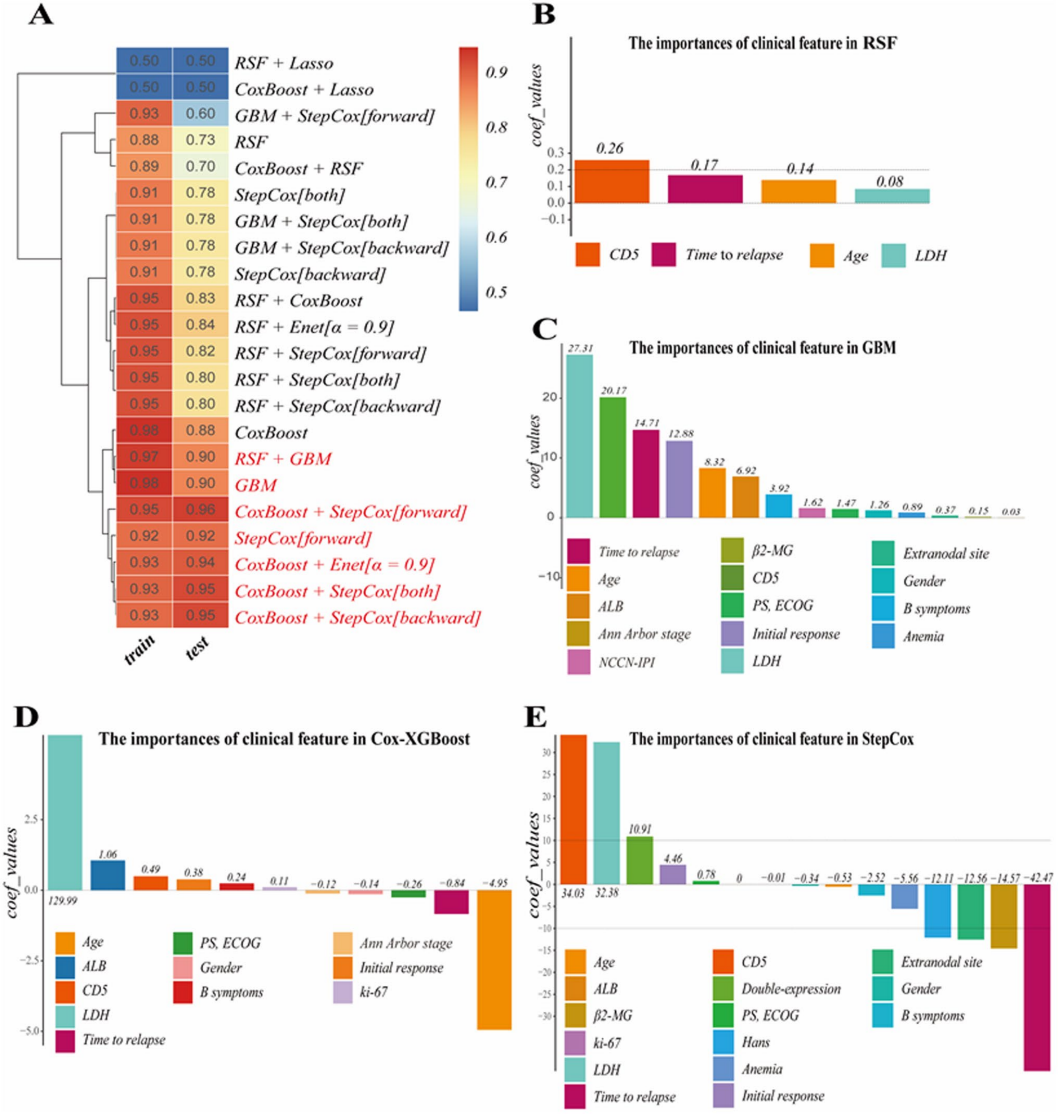
Machine learning framework for OS prediction. **A** Bootstrap-adjusted C-index of prognostic models. Variable importance rankings from: **B** RSF, **C** GBM, **D** Cox-XGBoost, **E** StepCox (forward)

**Fig. 3:**
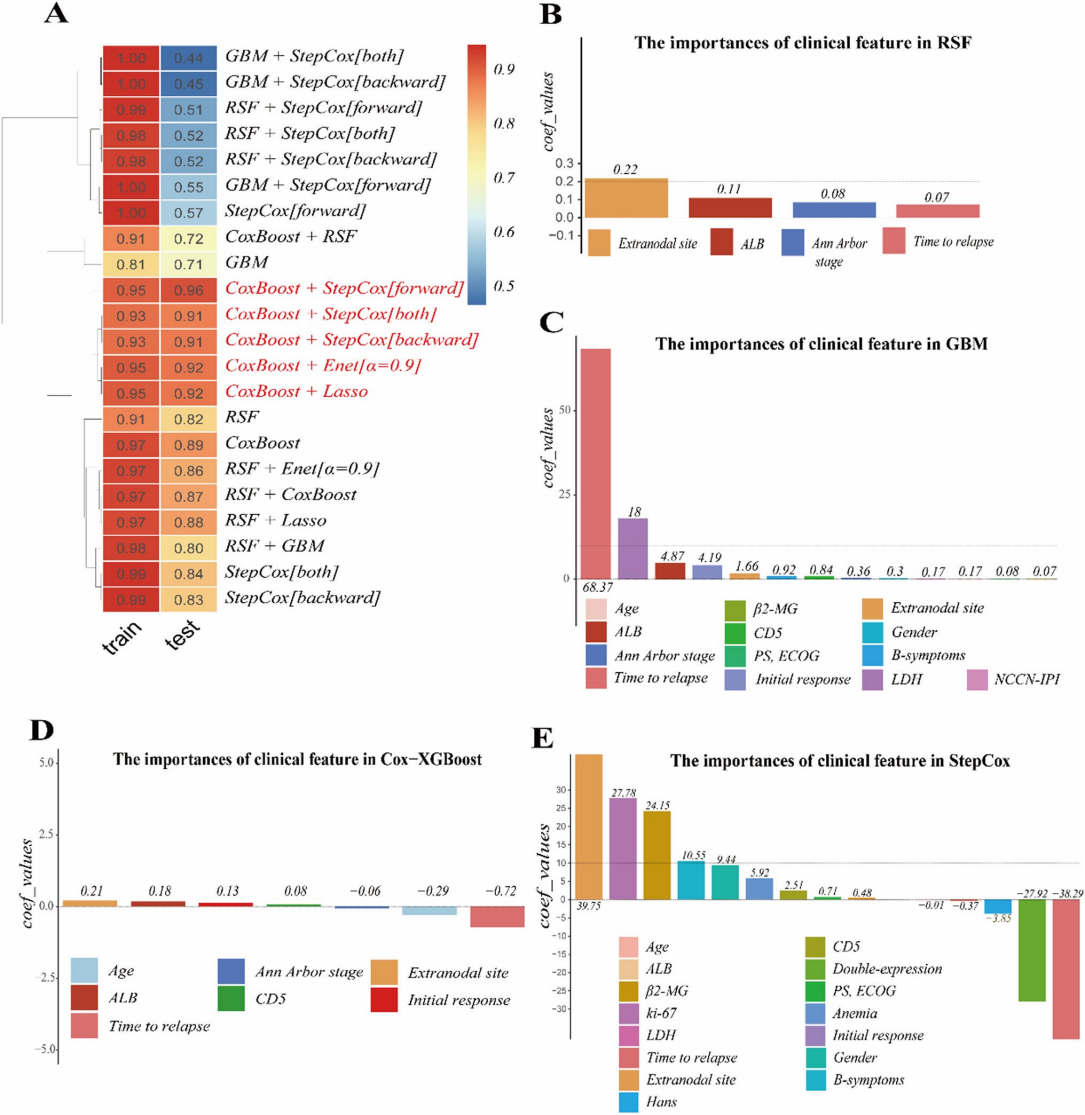
Machine learning framework for PFS prediction. **A** Bootstrap-adjusted C-index of prognostic models. Variable importance rankings from: **B** RSF, **C** GBM, **D** Cox-XGBoost, **E** StepCox (forward)

In this study, we utilized predictive models and subsequently computed the C-index for each model in relation to OS and PFS (Fig. 2A, Fig. 3A). Following 1000 bootstrap resamples, the adjusted C-index was 0.70.and red (representing coef values ≥ 0.9) in both the training and testing datasets. Harrell’s C-index is a discrimination metric in the range of 0.5 (no predictive power) to 1, with a value of 1 (perfect separation between event and non-event groups), Harrell’s C was weighted by inverse probability of censoring for the competing risks regression and machine learning models [14]. Notably, the optimal model was determined to be a combination of Cox proportional hazards model boosting (CoxBoost) and stepwise Cox (StepCox) (forward), which exhibited the highest average C-index (0.955). To identify key features associated with OS and PFS, the extreme gradient boosting Cox partial likelihood (Cox-XGBoost), generalized boosted regression modeling (GBM), random survival forest (RSF), and StepCox with bidirectional selection (both, backward, forward) models were trained. These models generated a bar chart where each bar represents a feature, and the height of the bar indicates its importance. Dotted lines were also included in the graph to facilitate the visualization of the impact of the features on the model results.

### The importance of clinical feature of OS

The importance of clinical features of OS in RSF is the CD5 + expression, time to relapse, age, and elevated LDH (Fig. 2B). For GBM, they are elevated LDH, CD5 + expression, time to relapse > 12 m, initial response, age, ALB, B symptoms, and anemia (Fig. 2C). In the Cox-XGBoost model, the key features are elevated LDH, ALB, CD5 + expression, time to relapse > 12 m, B symptoms, initial response, age (Fig. 2D). The StepCox model identifies CD5 + expression, LDH, double-expression, initial response, time to relapse > 12 m, β2-MG, extranodal site involvement, and Ann Arbor stage as significant (Fig. 2E).

### The importance of clinical feature of PFS

Regarding PFS, the RSF model highlights extranodal site involvement, ALB, Ann Arbor stage, and time to relapse > 12 m as important features (Fig. 3B). In the GBM model, time to relapse > 12 m, LDH, ALB, initial response, and extranodal site involvement are significant (Fig. 3C). The Cox-XGBoost model emphasizes extranodal site involvement, ALB, initial response, and time to relapse > 12 m (Fig. 3D). Finally, in the StepCox model, the significant features are extranodal site involvement, Ki-67, β2-MG, B symptoms, gender, time to relapse > 12 m, double-expression, and Hans classification (Fig. 3E).

The demonstrated significance of clinical features in OS and PFS supports the use of CoxBoost as an effective method for variable selection. Subsequent steps following the selection of variables via CoxBoost include the development of DCA, prognostic calibration curves, time-dependent ROC curves, and survival curves. Independent variables with a significance level of *P* < 0.05 should be included in the subsequent DCA analysis. The final competing risks regression model included seven predictors: PS, ECOG, B symptoms, LDH, ALB, initial response, time to relapse > 12 m, and CD5 + .

### Decision curve analysis of OS and PFS

The survival package was used for fitting the prognostic model, and the stdca.R file was used for DCA analysis. In the testing set, DCA showed that elevated LDH, initial response, time to relapse > 12 m, and CD5 + expression were associated with the best net benefit for OS and PFS. In the training set, elevated LDH, ALB, PS, ECOG = 2, initial response, and CD5 + expression were associated with the best net benefit for OS, while elevated LDH, initial response, CD5 + , time to relapse > 12 months, PS,ECOG = 1, PS, ECOG = 3, and B symptoms were associated with the best net benefit for PFS (Suppl table S1-2; Suppl Fig. S1).

### Prognostic calibration curves of OS and PFS

Calibration plots are essential tools for evaluating the agreement between predicted probabilities and observed outcomes across various percentiles of predicted values, typically displayed in deciles. Each repeated sampling involves a sample size of 25, with a total of 200 samplings. In the training set, the C-index for OS reached 0.932 (95% CI: 0.909–0.954), while for PFS, it was 0.972 (95% CI: 0.956–0.987). The calibration curve ( Suppl Fig. S2A, S2B) illustrates the calibration by evaluating the agreement between predicted probabilities and observed 1-year, 1.5-year, and 2-year survival rates. This plot offers valuable insights into the degree of alignment between the predicted probabilities of the model and the actual survival rates over time, thereby enhancing our comprehension of the model's performance.

### Time-dependent ROC curves of OS and PFS

The time-dependent ROC analysis demonstrated that CD5 + exhibited superior accuracy for OS and LDH for PFS. Specifically, the AUC values for predicting OS at 1, 2, and 3 years in the testing set, for OS the AUC values were 0.863, 0.864, and 0.898, while for PFS were 0.784, 0.769, and 0.776. In the training set, for predicting OS were 0.894, 0.836, and 0.860, and for PFS were 0.847, 0.848, and 0.869 (Suppl Fig. S2C, S2D, S2E, S2F).

### Treatment response and survival validation in clinical cohorts

**Fig. 4:**
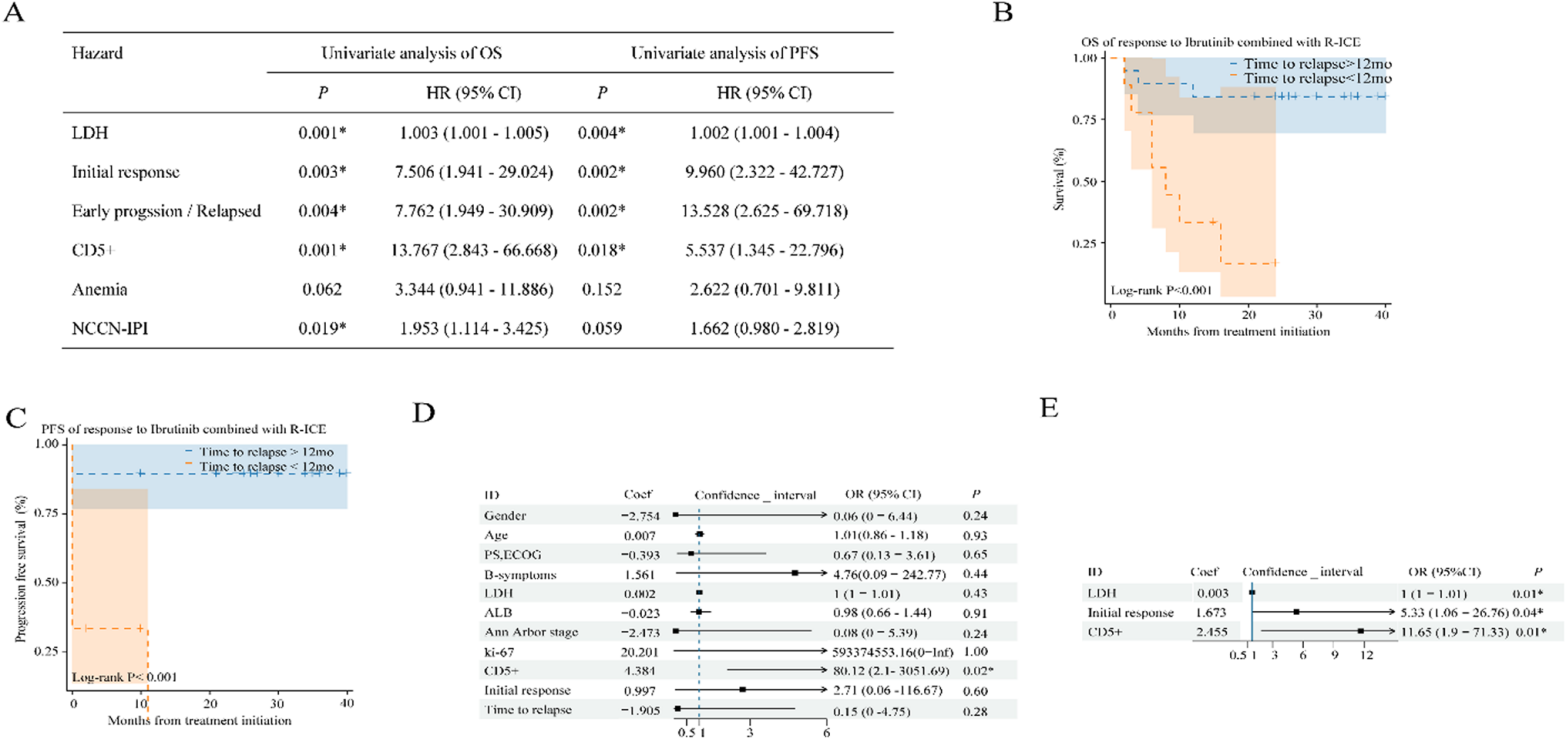
Survival outcomes in DLBCL patients treated with Ibrutinib plus R-ICE. A: Univariate analysis of PFS/OS in the clinical in-house cohort, B (OS) and C (PFS) illustrates the differences in time to relapse. Multivariable StepCox (forward) regression analysis of OS (D) and PFS (E) in the training set Note: HR: Hazard Ratio, OR: Odds Ratio,* Significance was indicated in two-sided p values < 0.05

In the clinical in-house cohort, an analysis was performed to evaluate the therapeutic efficacy and validate survival outcomes. Univariate analysis demonstrated that patients with elevated LDH levels (*P* = 0.001), an initial response characterized by stable disease or progression (*P* = 0.003), early progression or relapse (defined as time to relapse < 12 months) (*P* = 0.004), CD5 + expression (*P* = 0.001), and NCCN-IPI score (*P* = 0.019) were statistically significant predictors of OS. In terms of PFS, significant predictors included elevated LDH levels (*P* = 0.004), initial response as stable disease or progression (*P* = 0.002), early progression or relapse (*P* = 0.002), and CD5 + expression (*P* = 0.018). Patients with “time to relapse > 12 months” had better OS and PFS when treated with ibrutinib combined with R-ICE compared to those with “time to relapse > 12 months” (Fig. 4A, 4B, 4C).

### Multivariable Cox regression analysis of OS and PFS in the training set

In the training set, a multivariable StepCox (forward) regression analysis was con ducted to control for confounding variables, including age, gender, ECOG, PS, B symptoms, LDH, ALB, Ann Arbor stage, Ki-67 ≥ 70%, CD5 + , initial response, and time to relapse. The results of this analysis demonstrated that CD5 + (*P* = 0.02) remained statistically significant for OS, while LDH (*P* = 0.01), Initial response (*P* = 0.04), and CD5 + (*P* = 0.01) remained statistically significant for PFS (Fig. 4D, 4E).

### Safety evaluation

Among the patient cohort, a majority of 26 individuals (92.86%) experienced grade ≥ 3 adverse events. The incidence rates of leukopenia neutropenia, anemia, and thrombocytopenia were found to be 78.57%, 53.57%, and 92.86%, respectively. Nevertheless, no serious adverse events (SAEs) per CTCAE v5.0 criteria associated with the experimental drug were observed. Regrettably, one patient succumbed to pulmonary aspergillus, while another patient passed away due to septic shock caused by carbapenem-resistant Enterobacteriaceae septic shock. All the remaining patients demonstrated improvement following the administration of symptomatic supportive care.

## Discussion

The hematology field is witnessing growing interest in ML applications, though most ML studies remain retrospective analyses of electronic health records from single or multicenter databases [21]. Due to the limited amount of original clinical data and observation time, it was not possible to realize the prognostic model of PFS and multivariable Cox regression. Instead, we implemented an ML pipeline on a limited cohort of patients with baseline clinical variables to evaluate prognostic models for OS and PFS. CoxBoost with StepCox (forward) emerged as the optimal model for both OS and PFS. In training and testing set, CD5 + , initial response, time to relapse > 12 months, and elevated LDH emerged as crucial prognostic determinants. DCA showed that elevated LDH, initial response, and CD5 + were associated with the best net benefit for PFS and OS in the training and testing set. The C-index of prognostic calibration curve for OS was 0.932 (95% CI: 0.909–0.954), while for PFS, it was 0.972 (95% CI: 0.956–0.987). The time-dependent ROC analysis demonstrated that CD5 + exhibited superior accuracy for OS and elevated LDH for PFS. Univariate analysis revealed significant associations between early progression/relapse, CD5 + , elevated LDH, and prognosis in the testing set. Furthermore, multivariable Cox regression confirmed CD5 + as an independent OS preditor, with elevated LDH, initial response, and CD5 + retaining significance for PFS in the training set. High LDH levels may indicate a more aggressive disease and have been linked to poorer PFS and OS in patients receiving tisagenlecleucel [22]. Patients who receive rituximab with anthracycline-based immunochemotherapy in clinical trials and survive without progression after 24 months have similar survival rates to the general population for up to 7 years [23]. Most de novo CD5 + DLBCL patients are in the ABC subtype, have higher IPI, more extranodal disease, including CNS involvement, and worse outcomes compared to CD5-DLBCL. Previous study shows that despite rituximab-containing chemotherapy, stem cell transplantation does not improve outcomes for most de novo CD5 + DLBCL patients [24]. Anemia was not studied in large patient cohorts used to create the IPI or NCCN-IPI, but it has been linked to high-risk DLBCL features. Lower hemoglobin levels were associated with higher mortality. Patients with Hgb below the mean but still within the normal range had inferior OS compared to the reference group [25].

Despite progress in the upfront treatment of DLBCL, patients still experience relapses. The therapeutic landscape for rel/ref DLBCL has evolved substantially in recent years, with regulatory approvals of novel antibody–drug conjugates (ADCs), targeted small-molecule inhibitors, and CAR-T therapies significantly improved in the last couple of years [26]. Currently, transplant-eligible rel/ref DLBCL patients are typically treated with intensive salvage regimens, followed by HDT/ASCT rescue. However, many patients are ineligible for ASCT due to advanced age, comorbidities, or organ dysfunction [26, 27]. While CAR-T therapies have revolutionized outcomes in rel/ref DLBCL, only 30%-40% achieve durable remissions [28]. Furthermore, many rel/ref DLBCL patients are unfit for CAR-T therapies due to comorbidities or logistical constraints. The decision of which second-line regimen to choose for rel/ref DLBCL is indeed a topic that demands further investigation.

Ibrutinib is an oral irreversible inhibitor of BTK, which plays a critical role in the oncogenic signal transduction pathway downstream of the B-cell antigen receptor in various B-cell malignancies. It was approved by FDA in several B-cell lymphomas for its favorable efficacy and good tolerance [29]. A multi-institutional analysis of 54 rel/ref DLBCL patients treated with ibrutinib monotherapy reported an ORR of 28%, with median PFS of 1.7 months for GCB and 3.0 months for non-GCB subtypes, suggesting limited efficacy of single-agent ibrutinib [9]. However, combining ibrutinib (up to 840 mg/day) with R-ICE demonstrated improved tolerability and efficacy: 90% ORR (50% CR) with no dose-limiting toxicities, preserved CD34 + hematopoietic progenitor cell mobilization, and metabolic CR in 100% of non-GCB patients completing ≥ 1 treatment cycle [9]. In patients age younger than 60 years, ibrutinib plus R-CHOP in untreated non-GCB DLBCL improved event-free survival, PFS, and OS with manageable safety and can significantly improve untreated non-GCB DLBCL co-expressing BCL2 and MYC genes [30, 31]. In addition, molecular analysis showed that patients with both myeloid differentiation primary response 88 (MYD88) and cluster of differentiation 79B (CD79B) mutations were mostly sensitive to ibrutinib [32]. Salvage therapies may serve as a bridge to ASCT, allogeneic hematopoietic stem cell transplantation, or CAR-T cells. With the development of technology, a great many targeted drugs (BCL2 inhibitor, the histone deacetylas inhibitor, immunmodulator lenalidomide, obinutuzumab, phosphatidylinositol 3-kinase inhibitor, etc.) emerge and benefit B-cell lymphoma patients. The categorization of DLBCL has been revolutionized by the discovery of genetic subtypes that are determined by concurrent patterns of genetic modifications [33–35].

Although our machine learning framework achieved promising discrimination (C-index > 0.9), the small sample size (n = 28) inherently limits statistical power. To address this, we employed SMOTE-PSM for data augmentation and bootstrap validation to reduce overfitting. These methods are well-established in scenarios with limited clinical data. Future studies should prioritize multicenter collaboration to validate our findings in larger, diverse populations.

## Conclusions

The explainable CoxBoost + StepCox framework demonstrated high prognostic accuracy (C-index = 0.955) in a limited cohort of rel/ref DLBCL patients receiving ibrutinib plus R-ICE, and CD5 + expression, initial response, time to relapse > 12 months, and elevated LDH as independent prognostic biomarkers. This data-driven model demonstrates clinically translatable risk stratification capabilities, highlighting its potential to optimize treatment decision making for aggressive lymphoma. While promising, the model requires external validation through multicenter prospective studies to confirm generalizability. Furthermore, integrating longitudinal biomarker dynamics and real-world therapeutic sequencing data could enhance model adaptability, addressing the unmet need for precision prognostication in evolving therapeutic landscapes.

## Supplementary Information

Below is the link to the electronic supplementary material.

Supplementary file1 (DOCX 729 KB)

## Data Availability

No datasets were generated or analyzed during the current study.

## References

[1] Camicia R, Winkler HC, Hassa PO. Novel drug targets for personalized precision medicine in relapsed/refractory diffuse large B-cell lymphoma: a comprehensive review. Mol Cancer. 2015;14:207. 10.1186/s12943-015-0474-2. 26654227 10.1186/s12943-015-0474-2 PMC4676894

[2] Abramson JS, Solomon SR, Arnason J, et al. Lisocabtagene maraleucel as second-line therapy for large B-cell lymphoma: primary analysis of the phase 3 TRANSFORM study. Blood. 2023;141(14):1675–84. 10.1182/blood.2022018730. 36542826 10.1182/blood.2022018730 PMC10646768

[3] Philip T, Guglielmi C, Hagenbeek A, et al. Autologous bone marrow transplantation as compared with salvage chemotherapy in relapses of chemotherapy-sensitive non-Hodgkin’s lymphoma. N Engl J Med. 1995;333(23):1540–5. 10.1056/nejm199512073332305. 7477169 10.1056/NEJM199512073332305

[4] JACOBSON C A, MUNOZ J, SUN F, et al. Real-World Outcomes with Chimeric Antigen Receptor T Cell Therapies in Large B Cell Lymphoma: A Systematic Review and Meta-Analysis. Transplant Cell Ther, 2024, 30(1): 77.e1-.e15.10.1016/j.jtct.2023.10.017 10.1016/j.jtct.2023.10.017 37890589

[5] Roschewski M, Staudt LM, Wilson WH. Diffuse large B-cell lymphoma-treatment approaches in the molecular era. Nat Rev Clin Oncol. 2014;11(1):12–23. 10.1038/nrclinonc.2013.197. 24217204 10.1038/nrclinonc.2013.197 PMC7709161

[6] Wilson WH, Young RM, Schmitz R, et al. Targeting B cell receptor signaling with ibrutinib in diffuse large B cell lymphoma. Nat Med. 2015;21(8):922–6. 10.1038/nm.3884. 26193343 10.1038/nm.3884 PMC8372245

[7] Zheng X, Ding N, Song Y, et al. Different sensitivity of germinal center B cell-like diffuse large B cell lymphoma cells towards ibrutinib treatment. Cancer Cell Int. 2014;14(1):32. 10.1186/1475-2867-14-32. 24693884 10.1186/1475-2867-14-32 PMC3984027

[8] Kewalramani T, Zelenetz AD, Nimer SD, et al. Rituximab and ICE as second-line therapy before autologous stem cell transplantation for relapsed or primary refractory diffuse large B-cell lymphoma. Blood. 2004;103(10):3684–8. 10.1182/blood-2003-11-3911. 14739217 10.1182/blood-2003-11-3911

[9] Sauter CS, Matasar MJ, Schoder H, et al. A phase 1 study of ibrutinib in combination with R-ICE in patients with relapsed or primary refractory DLBCL. Blood. 2018;131(16):1805–8. 10.1182/blood-2017-08-802561. 29386196 10.1182/blood-2017-08-802561 PMC5909762

[10] Haug CJ, Drazen JM. Artificial Intelligence and Machine Learning in Clinical Medicine, 2023. N Engl J Med. 2023;388(13):1201–8. 10.1056/NEJMra2302038. 36988595 10.1056/NEJMra2302038

[11] Koivu A, Sairanen M, Airola A, et al. Synthetic minority oversampling of vital statistics data with generative adversarial networks. J Am Med Inform Assoc. 2020;27(11):1667–74. 10.1093/jamia/ocaa127. 32885818 10.1093/jamia/ocaa127 PMC7750982

[12] Vetter TR, Schober P, Mascha EJ. Diagnostic Testing and Decision-Making: Beauty Is Not Just in the Eye of the Beholder. Anesth Analg. 2018;127(4):1085–91. 10.1213/ane.0000000000003698. 30096083 10.1213/ANE.0000000000003698 PMC6135476

[13] Kamarudin AN, Cox T, Kolamunnage-Dona R. Time-dependent ROC curve analysis in medical research: current methods and applications. BMC Med Res Methodol. 2017;17(1):53. 10.1186/s12874-017-0332-6. 28388943 10.1186/s12874-017-0332-6 PMC5384160

[14] Clift AK, Collins GS, Lord S, et al. Predicting 10-year breast cancer mortality risk in the general female population in England: a model development and validation study. Lancet Digit Health. 2023;5(9):e571–81. 10.1016/s2589-7500(23)00113-9. 37625895 10.1016/S2589-7500(23)00113-9

[15] Vickers AJ, Elkin EB. Decision curve analysis: a novel method for evaluating prediction models. Med Decis Making. 2006;26(6):565–74. 10.1177/0272989x06295361. 17099194 10.1177/0272989X06295361 PMC2577036

[16] Huber M, Schober P, Petersen S, et al. Decision curve analysis confirms higher clinical utility of multi-domain versus single-domain prediction models in patients with open abdomen treatment for peritonitis. BMC Med Inform Decis Mak. 2023;23(1):63. 10.1186/s12911-023-02156-w. 37024840 10.1186/s12911-023-02156-w PMC10078078

[17] Hans CP, Weisenburger DD, Greiner TC, et al. Confirmation of the molecular classification of diffuse large B-cell lymphoma by immunohistochemistry using a tissue microarray. Blood. 2004;103(1):275–82. 10.1182/blood-2003-05-1545. 14504078 10.1182/blood-2003-05-1545

[18] Ananthamurthy A. An immunohistochemical study of double-expressor lymphomas and its correlation with cell of origin. J Cancer Res Ther. 2023;19(Supplement):S0. 10.4103/jcrt.jcrt_587_21. 37147953 10.4103/jcrt.jcrt_587_21

[19] Wen Q, Gao L, Xiong JK, et al. High-dose chemotherapy combined with autologous hematopoietic stem cell transplantation as frontline therapy for intermediate/high-risk diffuse large B cell lymphoma. Curr Med Sci. 2021;41(3):465–73. 10.1007/s11596-021-2394-2. 34218355 10.1007/s11596-021-2394-2

[20] Zhou Z, Sehn LH, Rademaker AW, et al. An enhanced International Prognostic Index (NCCN-IPI) for patients with diffuse large B-cell lymphoma treated in the rituximab era. Blood. 2014;123(6):837–42. 10.1182/blood-2013-09-524108. 24264230 10.1182/blood-2013-09-524108 PMC5527396

[21] ZACCARIA G M, FERRERO S, HOSTER E, et al. A Clinical Prognostic Model Based on Machine Learning from the Fondazione Italiana Linfomi (FIL) MCL0208 Phase III Trial. Cancers (Basel), 2021, 14(1).10.3390/cancers14010188 10.3390/cancers14010188 PMC8750124 35008361

[22] Rabinovich E, Pradhan K, Sica RA, et al. Elevated LDH greater than 400 U/L portends poorer overall survival in diffuse large B-cell lymphoma patients treated with CD19 CAR-T cell therapy in a real world multi-ethnic cohort. Exp Hematol Oncol. 2021;10(1):55. 10.1186/s40164-021-00248-9. 34886908 10.1186/s40164-021-00248-9 PMC8656085

[23] Maurer MJ, Habermann TM, Shi Q, et al. Progression-free survival at 24 months (PFS24) and subsequent outcome for patients with diffuse large B-cell lymphoma (DLBCL) enrolled on randomized clinical trials. Ann Oncol. 2018;29(8):1822–7. 10.1093/annonc/mdy203. 29897404 10.1093/annonc/mdy203 PMC6096732

[24] Alinari L, Gru A, Quinion C, et al. De novo CD5+ diffuse large B-cell lymphoma: adverse outcomes with and without stem cell transplantation in a large, multicenter, rituximab treated cohort. Am J Hematol. 2016;91(4):395–9. 10.1002/ajh.24299. 26800311 10.1002/ajh.24299 PMC4877689

[25] Clausen MR, Maurer MJ, Ulrichsen SP, et al. Pretreatment hemoglobin adds prognostic information to the NCCN-IPI in patients with diffuse large B-cell lymphoma treated with anthracycline-containing chemotherapy. Clin Epidemiol. 2019;11:987–96. 10.2147/clep.S219595. 31814771 10.2147/CLEP.S219595 PMC6861518

[26] Frontzek F, Karsten I, Schmitz N, et al. Current options and future perspectives in the treatment of patients with relapsed/refractory diffuse large B-cell lymphoma. Ther Adv Hematol. 2022;13:20406207221103320. 10.1177/20406207221103321. 35785244 10.1177/20406207221103321 PMC9243592

[27] Moore DC, Eagers KA, Janes A, et al. Tafasitamab and lenalidomide for relapsed/refractory diffuse large B-cell lymphoma in a patient on chronic intermittent hemodialysis. J Oncol Pharm Pract. 2023;29(1):239–41. 10.1177/10781552221102318. 35585701 10.1177/10781552221102318

[28] SAWALHA Y. Relapsed/Refractory Diffuse Large B-Cell Lymphoma: A Look at the Approved and Emerging Therapies. J Pers Med, 2021, 11(12).10.3390/jpm11121345 10.3390/jpm11121345 PMC8708171 34945817

[29] PAL SINGH S, DAMMEIJER F, HENDRIKS R W. Role of Bruton's tyrosine kinase in B cells and malignancies. Mol Cancer, 2018, 17(1): 57.10.1186/s12943-018-0779-z 10.1186/s12943-018-0779-z PMC5817726 29455639

[30] Younes A, Sehn LH, Johnson P, et al. Randomized phase III trial of ibrutinib and rituximab plus cyclophosphamide, doxorubicin, vincristine, and prednisone in non-germinal center B-cell diffuse large B-cell lymphoma. J Clin Oncol. 2019;37(15):1285–95. 10.1200/jco.18.02403. 30901302 10.1200/JCO.18.02403 PMC6553835

[31] Johnson PWM, Balasubramanian S, Hodkinson B, et al. Clinical impact of ibrutinib plus R-CHOP in untreated DLBCL coexpressing BCL2 and MYC in the phase 3 PHOENIX trial. Blood Adv. 2023;7(10):2008–17. 10.1182/bloodadvances.2022009389. 36696540 10.1182/bloodadvances.2022009389 PMC10188634

[32] Jiang S, Qin Y, Gui L, et al. Genomic alterations and MYD88(MUT) variant mapping in patients with diffuse large B-cell lymphoma and response to ibrutinib. Target Oncol. 2020;15(2):221–30. 10.1007/s11523-020-00710-4. 32239385 10.1007/s11523-020-00710-4

[33] Luo C, Yu T, Young KH, et al. HDAC inhibitor chidamide synergizes with venetoclax to inhibit the growth of diffuse large B-cell lymphoma via down-regulation of MYC, BCL2, and TP53 expression. J Zhejiang Univ Sci B. 2022;23(8):666–81. 10.1631/jzus.B2200016. 35953760 10.1631/jzus.B2200016 PMC9381329

[34] Vo DN, Alexia C, Allende-Vega N, et al. NK cell activation and recovery of NK cell subsets in lymphoma patients after obinutuzumab and lenalidomide treatment. Oncoimmunology. 2018;7(4): e1409322. 10.1080/2162402x.2017.1409322. 29632722 10.1080/2162402X.2017.1409322 PMC5889292

[35] KONG W, SENDER S, TAHER L, et al. BTK and PI3K Inhibitors Reveal Synergistic Inhibitory Anti-Tumoral Effects in Canine Diffuse Large B-Cell Lymphoma Cells. Int J Mol Sci, 2021, 22(23).10.3390/ijms222312673 10.3390/ijms222312673 PMC8658042 34884478

