# Supplementary material for: Predicting the risk of ibrutinib in combination with R-ICE in patients with relapsed or refractory DLBCL using explainable machine learning algorithms
Source: Clin Exp Med. 2025 May 26;25(1):177. doi: 10.1007/s10238-025-01709-9 (PMC12106145; doi:10.1007/s10238-025-01709-9)
Supplement: Supplementary file 1 — Supplementary file1 (DOCX 729 KB) [file 10238_2025_1709_MOESM1_ESM.docx]

**Table S1** The best net benefit for OS and PFS in the testing set

|  | OS in the testing set | | | PFS in the testing set | | |
| --- | --- | --- | --- | --- | --- | --- |
|  | 95% CI | C-index | P | 95% CI | C-index | P |
| Elevated LDH | 1.001 - 1.005 | 0.770 (0.691 - 0.850) | 0.0013* | 1.001 - 1.004 | 0.778 (0.677 - 0.880) | 0.0037* |
| Initial response | 1.941 - 29.024 | 0.694 (0.624 - 0.764) | 0.0035* | 2.322 - 42.727 | 0.761 (0.678 - 0.845) | 0.0020* |
| Time to relapse >12m | 1.949 - 30.909 | 0.730 (0.656 - 0.804) | 0.0037* | 2.625 - 69.718 | 0.818 (0.741 - 0.895) | 0.0018* |
| CD5+ | 2.843 - 66.668 | 0.800 (0.745 - 0.854) | 0.0011* | 1.345 - 22.796 | 0.744 (0.660 - 0.829) | 0.0178* |

* Significance was indicated in two-sided p values <0.05

**Table S2** The best net benefit for OS and PFS in the training set

|  | OS in the training set | | | PFS in the training set | | |
| --- | --- | --- | --- | --- | --- | --- |
|  | 95% CI | C-index | P | 95% CI | C-index | P |
| Elevated LDH | 1.002 - 1.005 | 0.873 (0.835 - 0.911) | 3.7e-07* | 1.002 - 1.005 | 0.782 (0.706 - 0.859) | 3.95e-06* |
| ALB | 0.796 - 0.989 | 0.641 (0.583 - 0.700) | 0.0301* | - | - | - |
| PS, ECOG=2 | 1.173 - 7.935 | 0.717 (0.667 - 0.768) | 0.0222* | - | - | - |
| Initial response | 0.043 - 0.257 | 0.763 (0.727 - 0.799) | 7.35e-07* | 5.027 - 41.731 | 0.772 (0.718 - 0.827) | 7.4e-07* |
| CD5+ | 4.345 - 27.859 | 0.773 (0.738 - 0.808) | 4.22e-07* | 2.330 - 15.681 | 0.736 (0.679 - 0.793) | 0.0002* |
| Time to relapse >12m | - | - | - | 7.586 - 444.589 | 0.878 (0.841 - 0.916) | 9.19e-05* |
| PS, ECOG=1 | - | - | - | 0.054 - 0.511, | 0.795 (0.743 - 0.847) | 0.0017* |
| PS,ECOG=3 | - | - | - | 0.011 - 0.629 | 0.795 (0.743 - 0.847) | 0.0161* |
| B-symptoms | - | - | - | 1.154 - 7.051 | 0.622 (0.561 - 0.682) | 0.0231* |

* Significance was indicated in two-sided p values <0.05


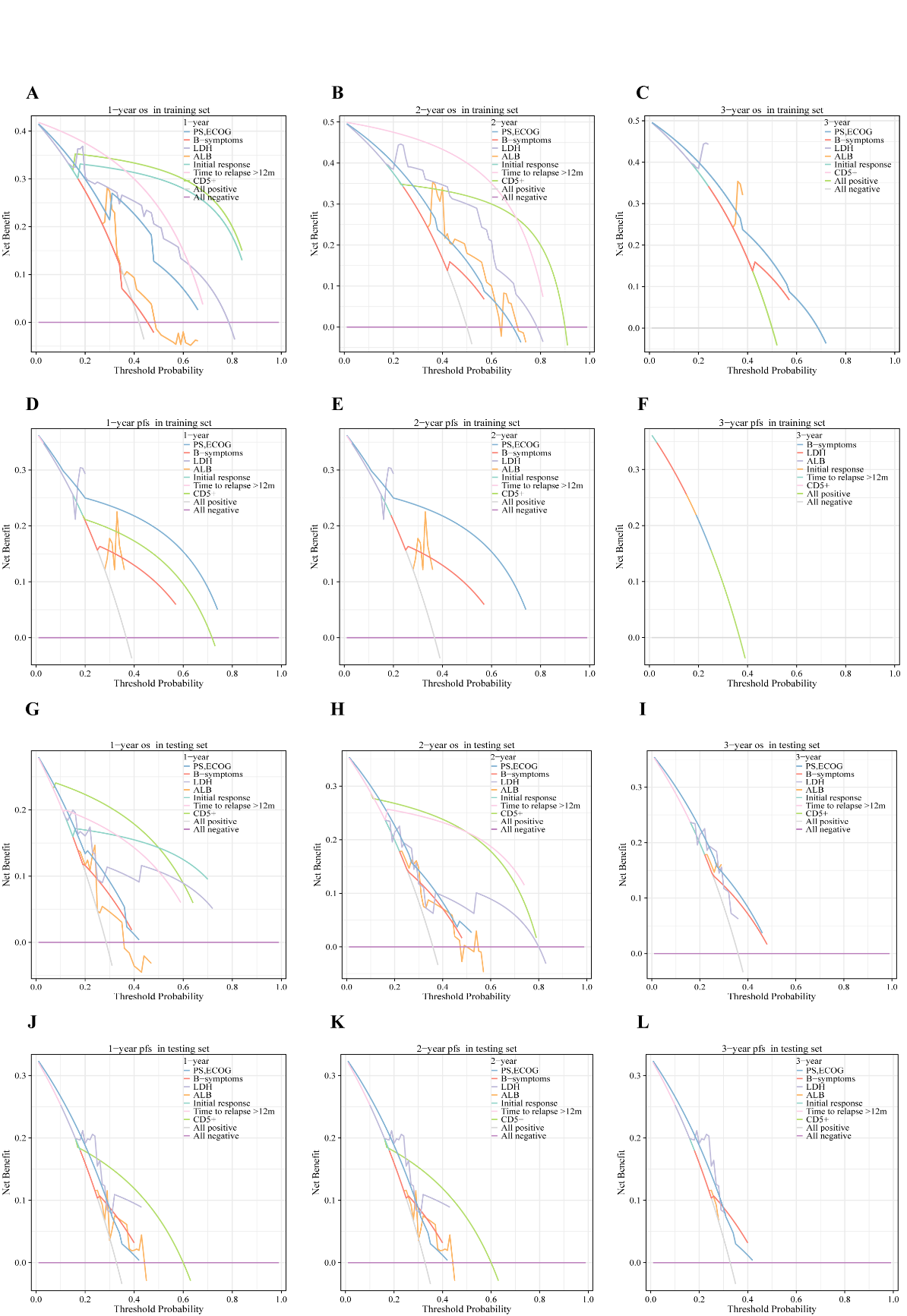


**Fig. S1** Clinical utility of each model assessed using net benefit


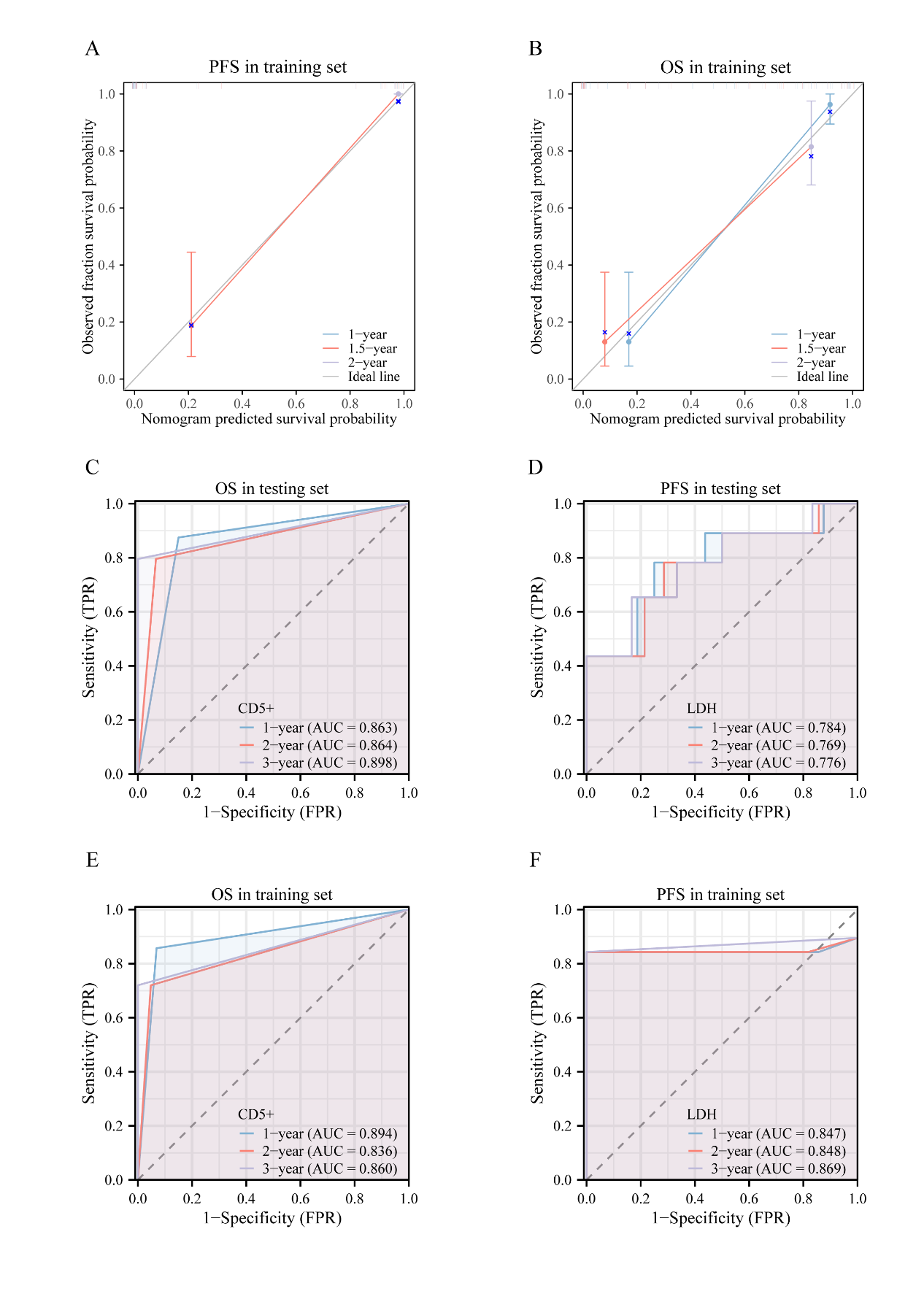


**Fig. S2** Prognostic Calibration Curves of OS and PFS and Time-dependent ROC analysis for predicting OS at 1, 2, and 3 years.
